# Practical Notes and Formulæ

**Published:** 1881-10-20

**Authors:** 


### PRACTICAL NOTES AND FORMULÆ.

**Dry Catarrh.**—In those conditions of the nasal membrane, where there is a tendency to accumulation of inspissated mucus, there is usually so much induration of the membrane, as to make it well-nigh insensible to ordinary vapors, or even medicated fluids. In such cases I have often been able to secure much comfort to my patients by having them use the following compound cubeb snuff:

| | | |
|---|---|---|
| R | Pulv. cubebæ | ℥j, |
| | Pulv. sacchari alb | Oj, |
| | Pulv. sanguinariæ | gr. lxxx, |
| | Pulv. g. camphoræ } | aa gr. L, |
| | Acidi carbolici, (cryst) } | |
| | Olei rosmarini | ♏ij, |
| | Olei gaultheriæ | ♏viij, |
| | M. ft. pulveris. | |

Sig. Use a small quantity, as a snuff, every three hours.—M. F. C. in *Medical Herald.*

**Painful Micturition.**—Dr. Bush, in Medical Times, says: Two inquiring friends ask, "What will relieve the severe pain caused by urinating during the acute stage of gonorrhœa?"

*Answer.*—The cause of this pain is both mechanical and chemical. The urethra is highly sensitive, and its caliber very much reduced, as a result of the inflammatory swelling; and the passage of a stream of urine forcibly dilating this inflamed and contracted canal, together with the action of the acid and irritating salts contained in the urine, combine to produce the pain. We may, in a great measure, overcome this difficulty by the use of the following formula, which acts as an antacid, anodyne and demulcent:

| | | |
|---|---|---|
| R | Tinct. hyosciami | ʒix, |
| | Potass. bi-carb | ʒij ss, |
| | Muscilag. acacia, q. s. | ℥vi. |

M. Sig. One tablespoonful every three hours.

**Anti-emetic.**—Dr. Randolph, in Medical Brief, suggests the following:

| | | |
|---|---|---|
| R | Creasote | 20 drops, |
| | Acet. acid | 40 drops, |
| | Morph. sulph | 2 grains, |
| | Aq. puræ | 2 ounces. |

M. Sig. Dose for an adult, teaspoonful in a little water.—*Chicago Med. Times.*

**Iodide of Potassium in Typhoid Fever.**—Writing in the Pacific Medical and Surgical Journal, for November, 1880, Dr. Oatman announces that iodide of potassium is "as much a specific in typhoid as quinine in intermittent fever." His exact plan is this:

An adult with uncomplicated typhoid fever, may take five grains of iodide of potassium every three hours, in a little sweetened water. Also every three hours one dessertspoonful of the following recipe, viz:

℞ Ol. terebinth ........................ } aa f. ʒi,

Tinct. anisi ........................ }

Vitel. ovi ............................ No. ij,

Sacchari· ............................ ʒij,

Aquæ puræ ad ........................ ℥ij.

M. Sig. This emulsion may be taken between the doses of the iodide.—*Med. and Surg. Rep.*

**Hemorrhoids.**—Dr. Todd, in the St. Louis Courier of Medicine, suggests, after a mild laxative, the following remedy for piles:

℞ Iodoform ............................ ʒi,

Balsam peru ............................ ʒii,

Cocoa butter and white wax, of each ............ ʒiss,

Calcined magnesia ............................ ʒi.

Incorporate the mass thoroughly, and divide into twelve suppositories. Insert one after each evacuation of the bowels, and oftener, if needed.—*Med. Herald.*

**"C. O. D."**—The Ohio Medical Journal gives the following story as told by a Dayton, Ohio, doctor who is perhaps a reader of the Michigan Medical News, from which he probably took his cue: The doctor was recently attending a case of labor in the family of one of his patrons, who, though a very excellent man, is a little slow in the payment of his medical bills. Immediately after the birth of the babe the father nervously asked: "Doctor, is the baby marked?" "Yes," quietly replied the doctor, "it is marked C. O. D." The hint was taken and the bill for that baby was promptly settled.—*Michigan Med. News.*

**Ointment for Old Sores.**—

℞ Chloral and iodoform, each ..................... ʒj,

Glycerine ............................ ʒij,

Ung. petrolii ............................ ʒvj.

Is recommended as an ointment to promote granulation in unhealthy ulcers.—*Med. Herald.*

**Luton's Exhilerant Mixture.**—Dr. Luton, of Reims, has found that the following mixture produces a highly exhilerating effect, somewhat similar to that of nitrous oxide, especially in excitable temperaments:

℞ Tincture of ergot ........................ 5 grams,
Sol. phosphate of soda (1-10) .............. 15 grams.

Take in a quarter of a glass of sugared water.

This produces "a lively gaiety and uncontrollable hilarity."—*Med. and Surg. Rep.*

**Tape Worm.**—Dr. McCormick suggests the following as an efficient remedy for tape worm. We have never failed with a pure article of kooso, but as this cannot always be obtained we annex the prescription.

The formula and directions are as follows :

℞ Castor oil.................................... ℥ j.

Spts. turpentine........................... ℥ j.

Tinct. myrrh .................................. ℥ j.

Worm seed oil................................ ʒ ij.

Croton oil ...................................... gtt. xx.

Mix.—Dose. Give from half a teaspoonful to a teaspoonful (according to the age of the child), with as much molasses, on an empty stomach, every half hour until two or three doses have been taken.—*Med. Herald.*

**Radical Cure of Rupture.**—The secret method of cure practiced by Dr. George Heaton successfully in one hundred and forty cases is now, after his death, published by Dr. J. H. Davenport. He injected extract of quercus alba into the hernial canal outside the peritoneal sac, to excite a mild degree of irritation in the tendons and fasciae, so as to lead to contraction. No fatal results followed nor any serious complications. It often cured, and when it failed great relief was obtained, so that a light truss sufficed to support the protrusion. —*Exchange.*

**Cure of Goiter by Fluoric Acid.**—Dr. Edward Woakes gives, in the Lancet, a detailed account of a number of cases of goiter cured by fluoric acid internally. He begins treatment with fifteen minims of a one-half-per-cent dilution of the acid three times a day, and, if necessary, increases the dose to twenty, thirty, forty, or even seventy minims, and extends the time to several months. His results are quite remarkable, even in cases that had resisted iodine, bromine, iron, etc. In a few it was conjoined with injections of tinct. iodine. Very few failed to be reasonably benefitted, and in eighty-five per cent the cure was decided.—*Med. News.*

**Chronic Ulcers.**—Dr. L. A. Davidson, of West Virginia, writes us that he cured an ulcer of 32 years' standing, by the application of the following—

℞ Acidi tannici.............................. ℥j,

Glycerinæ.................................... ʒiij,

Ext. pin. canad.............................. ʒiv. M.

To be applied on absorbent cotton.—*Med. and Surg. Rep.*

**Pruritus Vulvæ.**—A drachm to five ounces of warm water, (Braithwaite's Retrospect), is a good standard strength, but a stronger solution is usually needed, seldom a weaker. Hydrocyanic acid may be added—say ʒss of dilute acid to ℥x, or morphia (gr, ij), atropia (gr. ½), aconitia (gr. ½) or veratria (gr. ½). Infusion of tobacco (half an ounce to the pint) alone relieves some cases, and forms a good vehicle for borax or boracic acid.—*Chicago Med. Times.*

